# 4-Meth­oxy-*N*-(pyridin-4-ylmeth­yl)-3-(tri­fluoro­meth­yl)benzamide monohydrate

**DOI:** 10.1107/S1600536813029103

**Published:** 2013-10-31

**Authors:** S. Sreenivasa, N. R. Mohan, Vijith Kumar, B. S. Palakshamurthy, D. B. Arunakumar, P. A. Suchetan

**Affiliations:** a Department of Studies and Research in Chemistry, Tumkur University, Tumkur, Karnataka 572 103, India; b Solid State and Structural Chemistry Unit, Indian Institute of Science, Bangalore, India; c Department of Studies and Research in Physics, U.C.S., Tumkur University, Tumkur, Karnataka 572 103, India; d Department of Studies and Research in Chemistry, U.C.S., Tumkur University, Tumkur, Karnataka 572 103, India

## Abstract

In the title compound, C_15_H_13_F_3_N_2_O_2_·H_2_O, the dihedral angle between the benzene and pyridine rings is 74.97 (1)°. The –CF_3_ group attached to the benzene ring is *syn* to the C=O bond in the adjacent side chain. In the crystal, mol­ecules are linked to one another through the water mol­ecules by strong N—H⋯O, O—H⋯O and O—H⋯N hydrogen bonds, forming a ladder-type network. The benzamide mol­ecules are also linked to one another through C—H⋯F inter­actions, forming *C*(6) chains parallel to the *b-*axis direction. Aromatic π–π stacking inter­actions [centroid–centroid separations = 3.7150 (1) and 3.7857 (1) Å] between adjacent pairs of pyridine and benzene rings are also observed, resulting in a three-dimensional architecture are also observed.

## Related literature

For hydrogen-bond motifs, see: Bernstein *et al.* (1995 ▶). For the biological activity of amides, see: Manojkumar *et al.* (2013*a* ▶,*b* ▶); Sreenivasa *et al.* (2013*c* ▶). For the importance of amides containing tri­fluoro­methyl substituents as pharmacophores, see: Sreenivasa *et al.* (2013*a* ▶) and for amides providing structural rigidity to the mol­ecules, see: Sreenivasa *et al.* (2013*b* ▶).
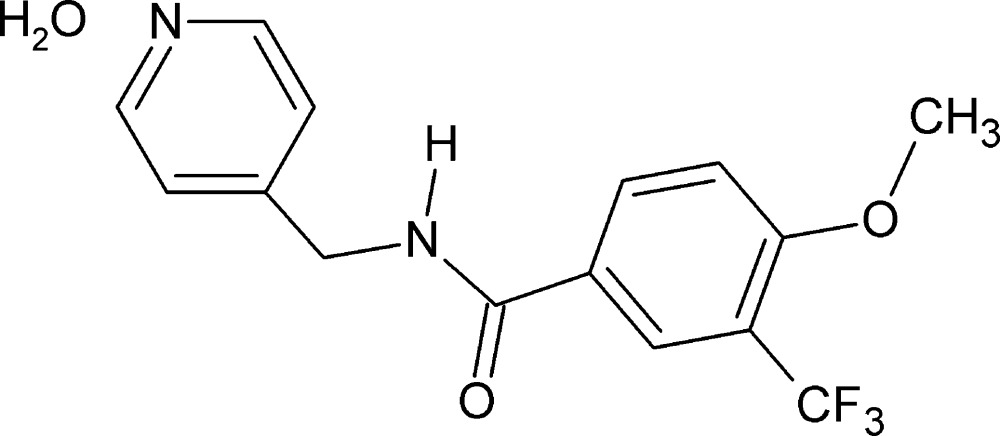


## Experimental

### 

#### Crystal data

C_15_H_13_F_3_N_2_O_2_·H_2_O
*M* *_r_* = 328.29
Triclinic, 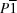

*a* = 7.2687 (13) Å
*b* = 7.8758 (14) Å
*c* = 14.177 (3) Å
α = 104.071 (10)°
β = 99.672 (10)°
γ = 97.21 (1)°
*V* = 764.2 (2) Å^3^
*Z* = 2
Mo *K*α radiation
μ = 0.12 mm^−1^
*T* = 296 K
0.34 × 0.28 × 0.22 mm

#### Data collection

Bruker APEXII CCD diffractometer
Absorption correction: ψ scan (*SADABS*; Bruker, 2009 ▶) *T*_min_ = 0.959, *T*_max_ = 0.973
11967 measured reflections
11967 independent reflections
9619 reflections with *I* > 2σ(*I*)

#### Refinement

*R*[*F* ^2^ > 2σ(*F* ^2^)] = 0.048
*wR*(*F* ^2^) = 0.135
*S* = 1.06
11967 reflections
212 parameters
H-atom parameters constrained
Δρ_max_ = 0.36 e Å^−3^
Δρ_min_ = −0.34 e Å^−3^

### 

Data collection: *APEX2* (Bruker, 2009 ▶); cell refinement: *APEX2* and *SAINT-Plus* (Bruker, 2009 ▶); data reduction: *SAINT-Plus* and *XPREP* (Bruker, 2009 ▶); program(s) used to solve structure: *SHELXS97* (Sheldrick, 2008 ▶); program(s) used to refine structure: *SHELXL97* (Sheldrick, 2008 ▶); molecular graphics: *Mercury* (Macrae *et al.*, 2008 ▶); software used to prepare material for publication: *SHELXL97*.

## Supplementary Material

Crystal structure: contains datablock(s) I, New_Global_Publ_Block. DOI: 10.1107/S1600536813029103/sj5360sup1.cif

Structure factors: contains datablock(s) I. DOI: 10.1107/S1600536813029103/sj5360Isup2.hkl

Supplementary material file. DOI: 10.1107/S1600536813029103/sj5360Isup3.cml

Additional supplementary materials:  crystallographic information; 3D view; checkCIF report

## Figures and Tables

**Table 1:** Hydrogen-bond geometry (Å, °)

*D*—H⋯*A*	*D*—H	H⋯*A*	*D*⋯*A*	*D*—H⋯*A*
N1—H*N*1⋯O3^i^	0.86	2.12	2.9119 (14)	152
O3—H1*O*⋯N2	0.85	2.01	2.8402 (15)	166
O3—H2*O*⋯O2^ii^	0.85	2.20	2.9646 (13)	149
C6—H6⋯F3^iii^	0.93	2.46	3.3963 (15)	173

Symmetry codes: (i) 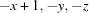; (ii) 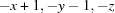; (iii) 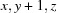.

**Fig. 1.**
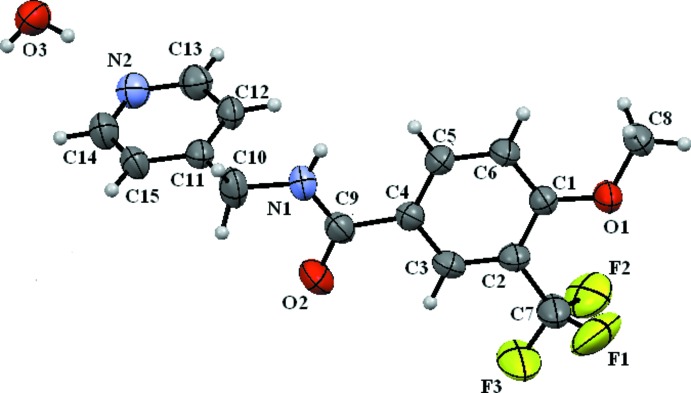
Molecular structure of the title compound, showing the atom-labeling scheme. Displacement ellipsoids are drawn at the 50% probability level.

**Fig. 2.**
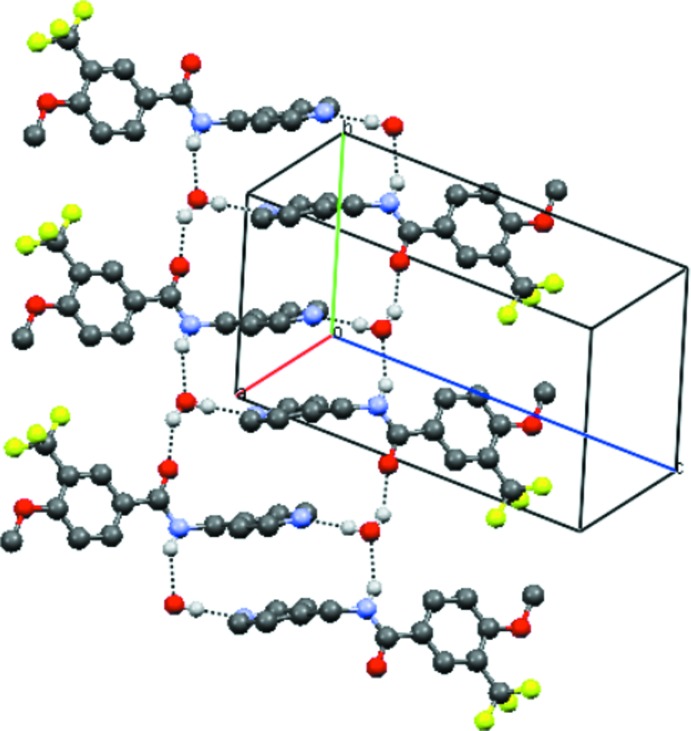
Linking of molecules in the crystal structure via water molecules, generating a ladder type network. H-atoms not involved in H-bonding are omitted.

**Fig. 3.**
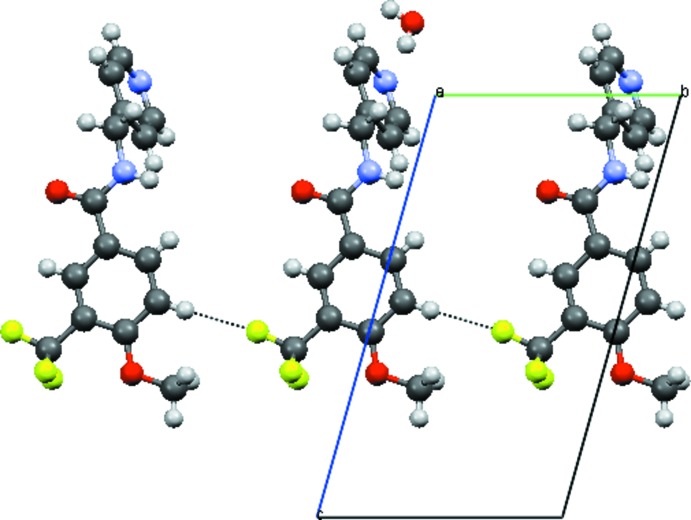
Linking of molecules into C(6) chains parallel to the b axis through C—H···F interactions.

**Fig. 4.**
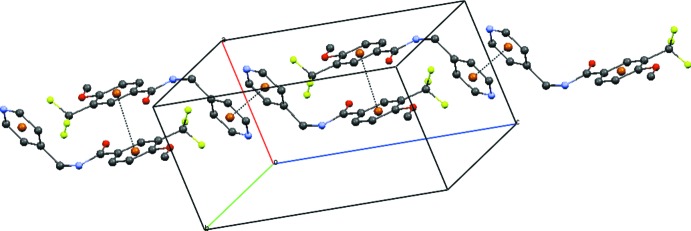
Aromatic π–π stacking interactions observed in the crystal structure.

## supplementary crystallographic information

### 1. Comment

Amides containing trifluoromethyl substituents have been considered as important pharmacophores (Sreenivasa *et al.* 2013*a*). Amide groups are very common in nature, form easily and provide structural rigidity to molecules (Sreenivasa *et al.* 2013*b*). Amides show a broad spectrum of pharmacological properties, including antibacterial (Manojkumar *et al.* 2013*a*), anti-inflammatory, antioxidant, analgesic and antiviral activity (Manojkumar *et al.* 2013*b*, Sreenivasa *et al.* 2013*c*). Keeping this in mind, the crystal structure of the title compound was determined.

In the title compound, C_15_H_13_F_3_N_2_O_2_·H_2_O, the dihedral angle between the benzene ring and the pyridine ring is 74.97 (1)°. The –CF_3_ group attached to the benzene ring is *syn* to the C=O bond in the adjacent side chain. Further, the conformation of the N—H bond in the chain is anti with respect to the C=O bond. In the crystal structure, the molecules are linked to one another *via* water molecules through strong N1—HN1···O3, O3—H2O···O2 and O3—H1O···N2 hydrogen bonds, forming a ladder type network. The benzamide molecules are also linked to one another forming C(6) chains (Bernstein *et al.*, 1995) parallel to the *b* axis through intermolecular C6—H6···F3 interactions. Further, aromatic π–π stacking interactions [centroid-centroid separations Cg1···Cg1 = 3.7150 (1) Å and Cg2···Cg2 = 3.7857 (1) Å] are also observed in the crystal structure. Cg1 and Cg2 are the centroids of the C11···C13,N2,C14,C15 and C1···C6 rings respectively.

### 2. Experimental

3-Tri-fluoromethyl-4-methoxy benzoic acid (1 mmol) and 1,1-carbonyldiimidazole (1.5 mmol) were dissolved in dichloroethane (5 ml) and heated to 45 *^o^*C for 30 min. 4-Aminomethyl pyridine (1.5 mmol) was added and the heating was continued for 4 h. The reaction was monitored by TLC. The organic layer was washed with sodium bicarbonate, dried using sodium sulfate and concentrated to yield the crude compound. This was further purified by column chromatography using petroleum ether / ethyl acetate (7:3) as eluent. Fine colorless crystals were grown by slow evaporation of the solvent system: petroleum ether / ethyl acetate (4:1) at room temperature.

### 3. Refinement

The hydrogen atoms attached to O3 were located in difference maps and refined in a rigid group. The remaining H atoms were positioned with idealized geometry using a riding model with N–H = 0.86 and C—H = 0.93 - 0.97 Å. The isotropic displacement parameters for all H atoms were set to 1.2 times *U*_eq_ of the parent atom or 1.5 times that of the parent atom for CH_3_.

### Crystal data

**Table:**

C_15_H_13_F_3_N_2_O_2_·H_2_O	*F*(000) = 340
*M**_r_* = 328.29	Prism
Triclinic, *P*1	*D*_x_ = 1.427 Mg m^−^^3^
Hall symbol: -P 1	Melting point: 485 K
*a* = 7.2687 (13) Å	Mo *K*α radiation, λ = 0.71073 Å
*b* = 7.8758 (14) Å	Cell parameters from 1167 reflections
*c* = 14.177 (3) Å	θ = 1.5–25.0°
α = 104.071 (10)°	µ = 0.12 mm^−^^1^
β = 99.672 (10)°	*T* = 296 K
γ = 97.21 (1)°	Prism, colourless
*V* = 764.2 (2) Å^3^	0.34 × 0.28 × 0.22 mm
*Z* = 2	

### Data collection

**Table:**

Bruker APEXII CCD diffractometer	11967 independent reflections
Radiation source: fine-focus sealed tube	9619 reflections with *I* > 2σ(*I*)
Graphite monochromator	*R*_int_ = 0.000
φ and ω scans	θ_max_ = 25.0°, θ_min_ = 1.5°
Absorption correction: ψ scan (*SADABS*; Bruker, 2009)	*h* = −8→8
*T*_min_ = 0.959, *T*_max_ = 0.973	*k* = −9→9
11967 measured reflections	*l* = −16→16

### Refinement

**Table:**

Refinement on *F*^2^	Primary atom site location: structure-invariant direct methods
Least-squares matrix: full	Secondary atom site location: difference Fourier map
*R*[*F*^2^ > 2σ(*F*^2^)] = 0.048	Hydrogen site location: inferred from neighbouring sites
*wR*(*F*^2^) = 0.135	H-atom parameters constrained
*S* = 1.06	*w* = 1/[σ^2^(*F*_o_^2^) + (0.0568*P*)^2^ + 0.2647*P*] where *P* = (*F*_o_^2^ + 2*F*_c_^2^)/3
11967 reflections	(Δ/σ)_max_ = 0.001
212 parameters	Δρ_max_ = 0.36 e Å^−^^3^
0 restraints	Δρ_min_ = −0.34 e Å^−^^3^

### Special details

**Table:**

Geometry. All s.u.'s (except the s.u. in the dihedral angle between two l.s. planes) are estimated using the full covariance matrix. The cell s.u.'s are taken into account individually in the estimation of s.u.'s in distances, angles and torsion angles; correlations between s.u.'s in cell parameters are only used when they are defined by crystal symmetry. An approximate (isotropic) treatment of cell s.u.'s is used for estimating s.u.'s involving l.s. planes.
Refinement. Refinement of *F*^2^ against ALL reflections. The weighted *R*-factor *wR* and goodness of fit *S* are based on *F*^2^, conventional *R*-factors *R* are based on *F*, with *F* set to zero for negative *F*^2^. The threshold expression of *F*^2^ > 2σ(*F*^2^) is used only for calculating *R*-factors(gt) *etc*. and is not relevant to the choice of reflections for refinement. *R*-factors based on *F*^2^ are statistically about twice as large as those based on *F*, and *R*- factors based on ALL data will be even larger.

### Fractional atomic coordinates and isotropic or equivalent isotropic displacement parameters (Å^2^)

**Table:**

	*x*	*y*	*z*	*U*_iso_*/*U*_eq_	
C4	0.20712 (13)	−0.16715 (12)	0.35758 (7)	0.0408 (2)	
C1	0.26262 (13)	0.00741 (12)	0.56032 (7)	0.0417 (3)	
C6	0.24913 (15)	0.10202 (13)	0.48940 (8)	0.0468 (3)	
H6	0.2589	0.2249	0.5092	0.056*	
C5	0.22150 (14)	0.01550 (13)	0.39008 (8)	0.0468 (3)	
H5	0.2123	0.0812	0.3437	0.056*	
C7	0.2577 (2)	−0.28518 (15)	0.60207 (9)	0.0678 (4)	
C3	0.22117 (14)	−0.26116 (13)	0.42910 (8)	0.0471 (3)	
H3	0.2120	−0.3840	0.4090	0.057*	
C11	0.32767 (14)	−0.24851 (13)	0.04242 (7)	0.0430 (3)	
C12	0.49099 (15)	−0.13759 (13)	0.09862 (7)	0.0471 (3)	
H12	0.4928	−0.0702	0.1626	0.057*	
C9	0.18106 (15)	−0.26938 (14)	0.25155 (8)	0.0488 (3)	
C2	0.24823 (14)	−0.17729 (13)	0.52871 (8)	0.0452 (3)	
C10	0.14376 (16)	−0.26534 (16)	0.07791 (8)	0.0605 (3)	
H10A	0.0539	−0.2148	0.0390	0.073*	
H10B	0.0931	−0.3906	0.0649	0.073*	
C13	0.65231 (16)	−0.12678 (14)	0.05948 (8)	0.0538 (3)	
H13	0.7607	−0.0502	0.0988	0.065*	
C8	0.30246 (17)	0.26947 (13)	0.69388 (8)	0.0567 (3)	
H8A	0.4111	0.3276	0.6766	0.085*	
H8B	0.3154	0.3032	0.7648	0.085*	
H8C	0.1901	0.3042	0.6635	0.085*	
C14	0.50371 (19)	−0.32623 (15)	−0.08476 (9)	0.0641 (3)	
H14	0.5059	−0.3927	−0.1484	0.077*	
C15	0.33680 (17)	−0.34444 (14)	−0.05187 (8)	0.0558 (3)	
H15	0.2301	−0.4212	−0.0929	0.067*	
N1	0.15688 (12)	−0.18044 (12)	0.18228 (6)	0.0520 (2)	
HN1	0.1489	−0.0696	0.2003	0.062*	
N2	0.66248 (14)	−0.21897 (12)	−0.03090 (7)	0.0586 (3)	
O1	0.28861 (11)	0.08100 (9)	0.65917 (5)	0.0556 (2)	
O3	0.89123 (14)	−0.17560 (10)	−0.17013 (7)	0.0713 (3)	
H1O	0.8411	−0.1809	−0.1207	0.107*	
H2O	0.8934	−0.2802	−0.2039	0.107*	
O2	0.18367 (14)	−0.43033 (10)	0.22891 (6)	0.0790 (3)	
F1	0.12080 (15)	−0.27319 (12)	0.65342 (7)	0.1170 (3)	
F2	0.41714 (13)	−0.23917 (11)	0.67010 (6)	0.1046 (3)	
F3	0.24329 (18)	−0.45486 (10)	0.55973 (6)	0.1459 (5)	

### Atomic displacement parameters (Å^2^)

**Table:**

	*U*^11^	*U*^22^	*U*^33^	*U*^12^	*U*^13^	*U*^23^
C4	0.0431 (6)	0.0365 (6)	0.0412 (6)	0.0039 (5)	0.0103 (5)	0.0078 (5)
C1	0.0483 (6)	0.0381 (6)	0.0362 (6)	0.0054 (5)	0.0060 (5)	0.0083 (5)
C6	0.0653 (7)	0.0322 (6)	0.0415 (7)	0.0077 (5)	0.0093 (5)	0.0091 (5)
C5	0.0609 (7)	0.0417 (6)	0.0386 (7)	0.0070 (5)	0.0096 (5)	0.0136 (5)
C7	0.1090 (11)	0.0452 (8)	0.0500 (8)	0.0187 (7)	0.0111 (8)	0.0155 (6)
C3	0.0574 (7)	0.0307 (6)	0.0511 (7)	0.0058 (5)	0.0106 (5)	0.0082 (5)
C11	0.0548 (6)	0.0382 (6)	0.0346 (6)	0.0066 (5)	0.0068 (5)	0.0092 (5)
C12	0.0563 (7)	0.0476 (6)	0.0328 (6)	0.0076 (5)	0.0047 (5)	0.0060 (5)
C9	0.0508 (6)	0.0430 (7)	0.0475 (7)	0.0026 (5)	0.0119 (5)	0.0045 (6)
C2	0.0566 (7)	0.0360 (6)	0.0444 (7)	0.0081 (5)	0.0089 (5)	0.0142 (5)
C10	0.0565 (7)	0.0739 (8)	0.0378 (7)	−0.0006 (6)	0.0064 (5)	−0.0013 (6)
C13	0.0532 (7)	0.0541 (7)	0.0490 (7)	0.0039 (5)	0.0024 (6)	0.0124 (6)
C8	0.0814 (8)	0.0411 (6)	0.0421 (7)	0.0100 (6)	0.0077 (6)	0.0048 (5)
C14	0.0830 (9)	0.0553 (8)	0.0476 (7)	0.0037 (7)	0.0236 (7)	−0.0009 (6)
C15	0.0655 (8)	0.0478 (7)	0.0425 (7)	−0.0041 (6)	0.0120 (6)	−0.0029 (5)
N1	0.0636 (6)	0.0506 (5)	0.0366 (5)	0.0064 (4)	0.0126 (4)	0.0026 (4)
N2	0.0638 (6)	0.0549 (6)	0.0572 (7)	0.0086 (5)	0.0185 (5)	0.0120 (5)
O1	0.0865 (6)	0.0409 (4)	0.0364 (4)	0.0108 (4)	0.0067 (4)	0.0091 (3)
O3	0.0933 (6)	0.0590 (5)	0.0690 (6)	0.0117 (5)	0.0364 (5)	0.0185 (4)
O2	0.1277 (8)	0.0419 (5)	0.0609 (6)	0.0138 (5)	0.0235 (5)	−0.0004 (4)
F1	0.1480 (8)	0.1277 (8)	0.1152 (7)	0.0268 (6)	0.0619 (7)	0.0820 (6)
F2	0.1252 (7)	0.1093 (7)	0.0871 (6)	0.0239 (5)	−0.0084 (5)	0.0600 (5)
F3	0.3145 (16)	0.0451 (5)	0.0806 (6)	0.0383 (6)	0.0229 (7)	0.0305 (4)

### Geometric parameters (Å, º)

**Table:**

C4—C5	1.3843 (13)	C12—H12	0.9300
C4—C3	1.3914 (13)	C9—O2	1.2331 (12)
C4—C9	1.4903 (14)	C9—N1	1.3392 (13)
C1—O1	1.3506 (12)	C10—N1	1.4495 (13)
C1—C6	1.3875 (14)	C10—H10A	0.9700
C1—C2	1.3989 (13)	C10—H10B	0.9700
C6—C5	1.3753 (14)	C13—N2	1.3295 (14)
C6—H6	0.9300	C13—H13	0.9300
C5—H5	0.9300	C8—O1	1.4305 (12)
C7—F3	1.3079 (13)	C8—H8A	0.9600
C7—F2	1.3240 (15)	C8—H8B	0.9600
C7—F1	1.3279 (16)	C8—H8C	0.9600
C7—C2	1.4929 (15)	C14—N2	1.3319 (15)
C3—C2	1.3746 (14)	C14—C15	1.3743 (16)
C3—H3	0.9300	C14—H14	0.9300
C11—C12	1.3750 (14)	C15—H15	0.9300
C11—C15	1.3832 (14)	N1—HN1	0.8600
C11—C10	1.5072 (15)	O3—H1O	0.8499
C12—C13	1.3808 (15)	O3—H2O	0.8500
			
C5—C4—C3	117.63 (9)	C3—C2—C1	119.91 (9)
C5—C4—C9	124.51 (9)	C3—C2—C7	119.49 (9)
C3—C4—C9	117.85 (9)	C1—C2—C7	120.59 (10)
O1—C1—C6	124.57 (9)	N1—C10—C11	115.26 (9)
O1—C1—C2	116.78 (8)	N1—C10—H10A	108.5
C6—C1—C2	118.65 (9)	C11—C10—H10A	108.5
C5—C6—C1	120.53 (9)	N1—C10—H10B	108.5
C5—C6—H6	119.7	C11—C10—H10B	108.5
C1—C6—H6	119.7	H10A—C10—H10B	107.5
C6—C5—C4	121.53 (9)	N2—C13—C12	124.12 (10)
C6—C5—H5	119.2	N2—C13—H13	117.9
C4—C5—H5	119.2	C12—C13—H13	117.9
F3—C7—F2	106.29 (11)	O1—C8—H8A	109.5
F3—C7—F1	105.41 (12)	O1—C8—H8B	109.5
F2—C7—F1	104.90 (11)	H8A—C8—H8B	109.5
F3—C7—C2	112.39 (10)	O1—C8—H8C	109.5
F2—C7—C2	113.54 (11)	H8A—C8—H8C	109.5
F1—C7—C2	113.58 (10)	H8B—C8—H8C	109.5
C2—C3—C4	121.74 (9)	N2—C14—C15	123.86 (11)
C2—C3—H3	119.1	N2—C14—H14	118.1
C4—C3—H3	119.1	C15—C14—H14	118.1
C12—C11—C15	116.65 (10)	C14—C15—C11	119.91 (11)
C12—C11—C10	123.31 (10)	C14—C15—H15	120.0
C15—C11—C10	120.02 (9)	C11—C15—H15	120.0
C11—C12—C13	119.61 (10)	C9—N1—C10	121.98 (10)
C11—C12—H12	120.2	C9—N1—HN1	119.0
C13—C12—H12	120.2	C10—N1—HN1	119.0
O2—C9—N1	121.38 (10)	C13—N2—C14	115.85 (10)
O2—C9—C4	120.76 (10)	C1—O1—C8	118.17 (7)
N1—C9—C4	117.85 (9)	H1O—O3—H2O	109.5
			
O1—C1—C6—C5	−179.63 (9)	F3—C7—C2—C3	1.81 (18)
C2—C1—C6—C5	0.23 (15)	F2—C7—C2—C3	122.49 (12)
C1—C6—C5—C4	−0.29 (16)	F1—C7—C2—C3	−117.75 (12)
C3—C4—C5—C6	0.15 (15)	F3—C7—C2—C1	−179.53 (12)
C9—C4—C5—C6	−178.73 (10)	F2—C7—C2—C1	−58.85 (15)
C5—C4—C3—C2	0.05 (15)	F1—C7—C2—C1	60.90 (15)
C9—C4—C3—C2	179.00 (9)	C12—C11—C10—N1	−10.41 (15)
C15—C11—C12—C13	0.16 (14)	C15—C11—C10—N1	171.11 (10)
C10—C11—C12—C13	−178.37 (9)	C11—C12—C13—N2	−0.43 (16)
C5—C4—C9—O2	174.51 (10)	N2—C14—C15—C11	−0.26 (18)
C3—C4—C9—O2	−4.36 (15)	C12—C11—C15—C14	0.16 (15)
C5—C4—C9—N1	−4.80 (15)	C10—C11—C15—C14	178.74 (10)
C3—C4—C9—N1	176.33 (9)	O2—C9—N1—C10	−3.73 (16)
C4—C3—C2—C1	−0.10 (16)	C4—C9—N1—C10	175.58 (9)
C4—C3—C2—C7	178.57 (10)	C11—C10—N1—C9	−93.86 (12)
O1—C1—C2—C3	179.83 (9)	C12—C13—N2—C14	0.33 (16)
C6—C1—C2—C3	−0.04 (15)	C15—C14—N2—C13	0.02 (17)
O1—C1—C2—C7	1.17 (15)	C6—C1—O1—C8	0.32 (15)
C6—C1—C2—C7	−178.69 (10)	C2—C1—O1—C8	−179.53 (9)

### Hydrogen-bond geometry (Å, º)

**Table:**

*D*—H···*A*	*D*—H	H···*A*	*D*···*A*	*D*—H···*A*
N1—H*N*1···O3^i^	0.86	2.12	2.9119 (14)	152
O3—H1*O*···N2	0.85	2.01	2.8402 (15)	166
O3—H2*O*···O2^ii^	0.85	2.20	2.9646 (13)	149
C6—H6···F3^iii^	0.93	2.46	3.3963 (15)	173

Symmetry codes: (i) −*x*+1, −*y*, −*z*; (ii) −*x*+1, −*y*−1, −*z*; (iii) *x*, *y*+1, *z*.

## References

[bb1] Bernstein, J., Davis, R. E., Shimoni, L. & Chang, N.-L. (1995). *Angew. Chem. Int. Ed. Engl.* **34**, 1555–1573.

[bb2] Bruker (2009). *APEX2*, *SADABS*, *SAINT-Plus* and *XPREP* Bruker AXS Inc., Madison, Wisconsin, USA.

[bb3] Macrae, C. F., Bruno, I. J., Chisholm, J. A., Edgington, P. R., McCabe, P., Pidcock, E., Rodriguez-Monge, L., Taylor, R., van de Streek, J. & Wood, P. A. (2008). *J. Appl. Cryst.* **41**, 466–470.

[bb4] Manojkumar, K. E., Sreenivasa, S., Mohan, N. R., Madhuchakrapani Rao, T. & Harikrishna, T. (2013*a*). *J. Appl. Chem.* **2**, 730–737.

[bb5] Manojkumar, K. E., Sreenivasa, S., Shivaraja, G. & Madhuchakrapani Rao, T. (2013*b*). *Molbank.* **M803**; 10.3390/M803.

[bb6] Sheldrick, G. M. (2008). *Acta Cryst.* A**64**, 112–122. 10.1107/S0108767307043930 18156677

[bb7] Sreenivasa, S., ManojKumar, K. E., Kempaiah, A., Suchetan, P. A. & Palakshamurthy, B. S. (2013*a*). *Acta Cryst.* E**69**, o761. 10.1107/S1600536813010131 PMC3648287 23723907

[bb8] Sreenivasa, S., ManojKumar, K. E., Suchetan, P. A., Tonannavar, J., Chavan, Y. & Palakshamurthy, B. S. (2013*b*). *Acta Cryst.* E**69**, o185. 10.1107/S1600536812051690 PMC3569247 23424470

[bb9] Sreenivasa, S., Palakshamurthy, B. S., Lohith, T. N., Mohan, N. R., Kumar, V. & Suchetan, P. A. (2013*c*). *Acta Cryst.* E**69**, o1263. 10.1107/S1600536813019107 PMC3793760 24109347

